# Supplementary material for: Identification and Expression Analysis of MPK and MKK Gene Families in Pecan (Carya illinoinensis)
Source: Int J Mol Sci. 2022 Dec 2;23(23):15190. doi: 10.3390/ijms232315190 (PMC9737717; doi:10.3390/ijms232315190)
Supplement: Supplementary file 1 [file ijms-23-15190-s001.zip › ijms-2048829-Supplementary files/Supplementary Figures.docx]

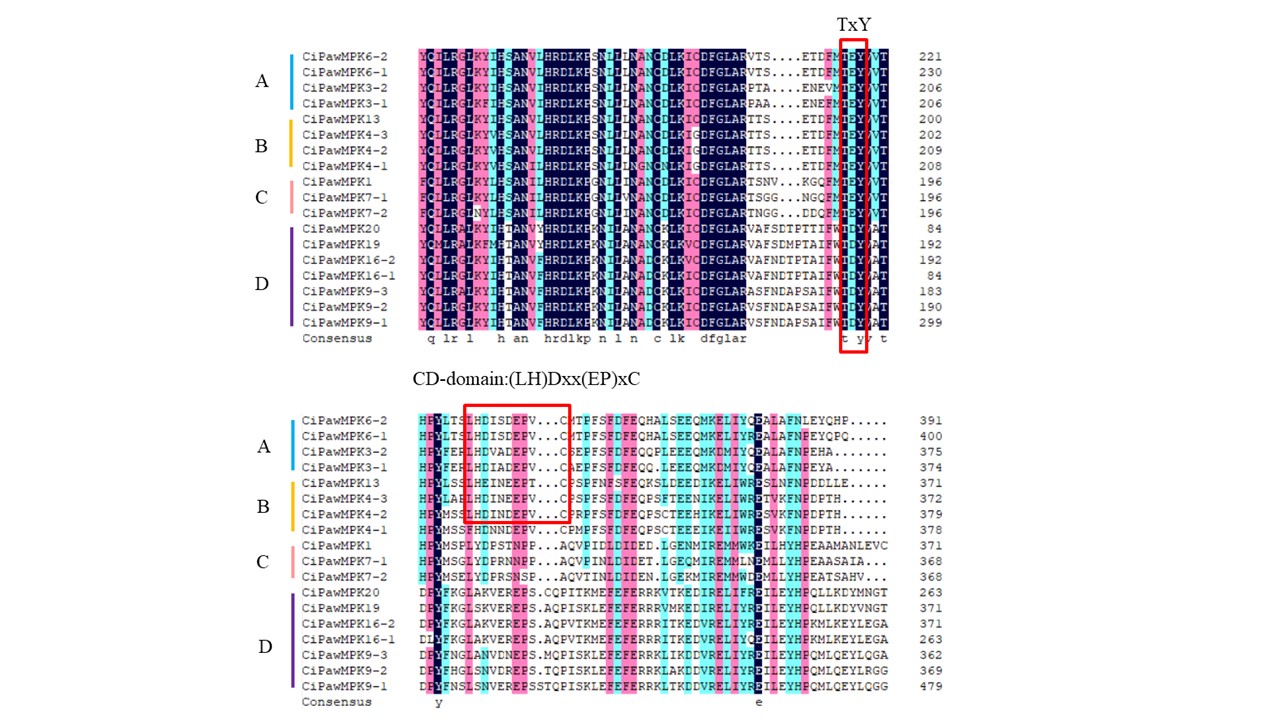


**Figure S1**. Multiple sequence alignment of MPK gene of pecan. The TxY (conserved N-terminal TEY motif and activation loop TDY motif) sequence specific to the MPK gene and the CD domain contained in the group A and B genes are circled in red.


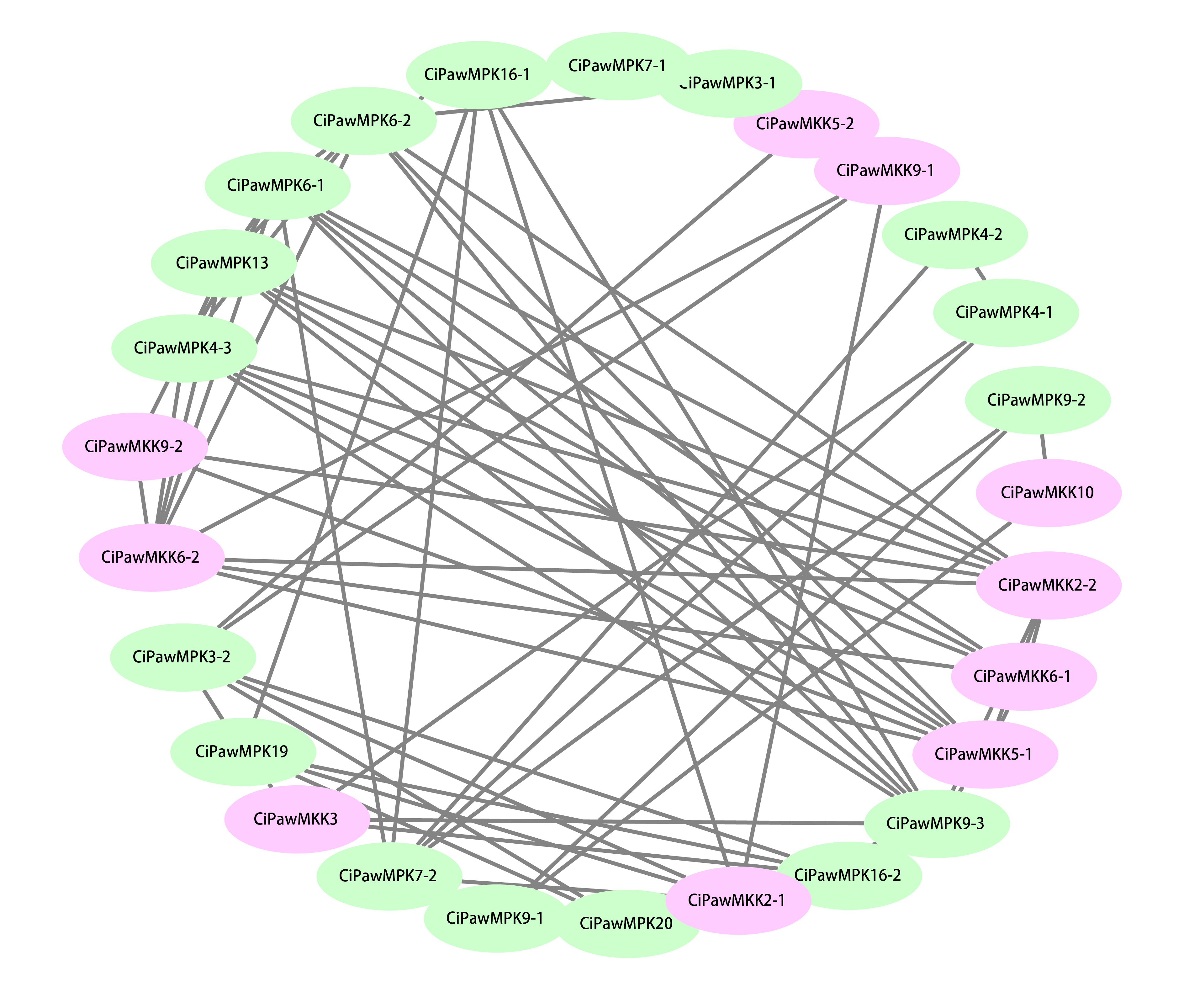


**Figure S2**. The co-expression network of pecan MPK and MKK genes, 27 nodes (17 MPK are represented by light purple ellipses and 10 MKK are represented by light green ellipses) and 43 edges were found, and 14 nodes contained more than 6 edges, indicating a strong correlation between these nodes.





**Figure S3.** Enzyme activity analysis of pecan. **(a)** SOD activity treated with NaCl, **(b)** SOD activity treated with 20％PEG, **(c)** POD activity treated with NaCl, **(d)** POD activity treated with 20％PEG. According to Duncan's multiple range test, it was judged whether there was a significant difference in the expression levels at different time points (P < 0.05), and the difference results were denoted by lowercase letters. Error bars represent mean ± SE obtained from three biological replicates.
